# *In vitro* evaluation of anti-hepatoma activity of brevilin A: involvement of Stat3/Snail and Wnt/β-catenin pathways

**DOI:** 10.1039/c8ra08574a

**Published:** 2019-02-05

**Authors:** Yaguang Qin, Hong Lu

**Affiliations:** Department of Oncology, Huaihe Hospital of Henan University No. 8 Baobei Road Kaifeng 475000 Henan PR China honglu6512@163.com +86037123906821

## Abstract

Brevilin A, a natural sesquiterpene lactone extracted from *Centipeda minima*, has been found to exhibit anti-tumor effect. However, the roles of brevilin A on hepatocellular carcinoma (HCC) have not yet been reported. The aim of the present study was to investigate the role of brevilin A in HCC and the underlying *in vitro* mechanisms. The HCC cell lines, HepG2 and SMMC-7221, were treated with different concentrations of brevilin A (0 μM, 2.5 μM, 5 μM, 10 μM, 15 μM, and 20 μM) for 48 h. MTT assay was performed to detect the cell viability. Flow cytometry was performed to detect cell apoptosis. Cell invasion was detected using the transwell assay. The expressions of matrix metalloproteinase (MMP)-2, MMP-9, phospho-signal transducer and activator of transcription 3 (p-Stat3), Stat3, Snail, β-catenin, and c-Myc were detected using the western blot analysis. The results showed that brevilin A reduced cell viability and invasion in HepG2 and SMMC-7221 cells. The apoptotic rates of HepG2 and SMMC-7221 cells treated with brevilin A were found to be markedly increased. The expression levels of MMP-2 and MMP-9 were decreased after the treatment with brevilin A. In addition, brevilin A suppressed the Stat3/Snail and Wnt/β-catenin signaling pathways in HCC cells. Collectively, brevilin A displayed an anti-tumor effect against HCC *in vitro*, which might be attributed to the inactivation of Stat3/Snail and Wnt/β-catenin signaling pathways.

## Abstract 2

Brevilin A, a natural sesquiterpene lactone extracted from *Centipeda minima*, has been found to exhibit anti-tumor effect.

## Graphical abstract

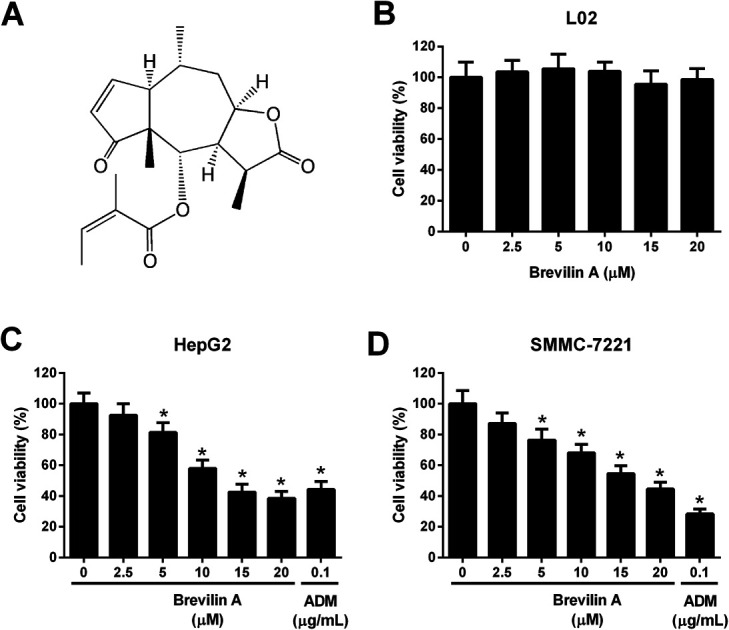

## 1. Introduction

Hepatocellular carcinoma (HCC) is one of the most common types of primary liver cancer in adults with the increasing incidence and death rates in recent years.^1,2^ The HCC remains the most frequent cause of death in patients with chronic liver diseases such as chronic HBV infection or cirrhosis.^3^ The common therapies for HCC include liver resection, local ablative therapies or liver transplantation; however, the 5 year survival rate for HCC patients remains poor.^3^ The therapies are more curative for early-stage patients (less than 10%) than advanced-stage HCC patients (about 50–80%).^2,4,5^ Therefore, the therapies against advanced states of HCC patients and high recurrence rates after surgical resection are urgently needed.

Understanding the molecular mechanism of the tumorigenesis is helpful in exploring new therapeutic approaches of HCC. Increasing studies have demonstrated that many signaling pathways are involved in the development and progression of HCC, such as PI3K/AKT, mitogen-activated protein kinase (MAPK), signal transducer and activator of transcription (STAT), and Wnt/β-catenin pathways.^6–8^ The constituents from *Picrasma quassioides* were reported to induce cell apoptosis of the HCC cell lines through the downregulation of MAPK/ERK pathway.^7^ The epidermal growth factor-like domain multiple 7 (EGFL7) promotes the cell proliferation and inhibits the apoptosis of HCC cell lines *via* activating the Wnt/β-catenin pathway.^6^ Therefore, targeting these signaling pathways might be potential therapies for the treatment of HCC.

**Fig. 1:**
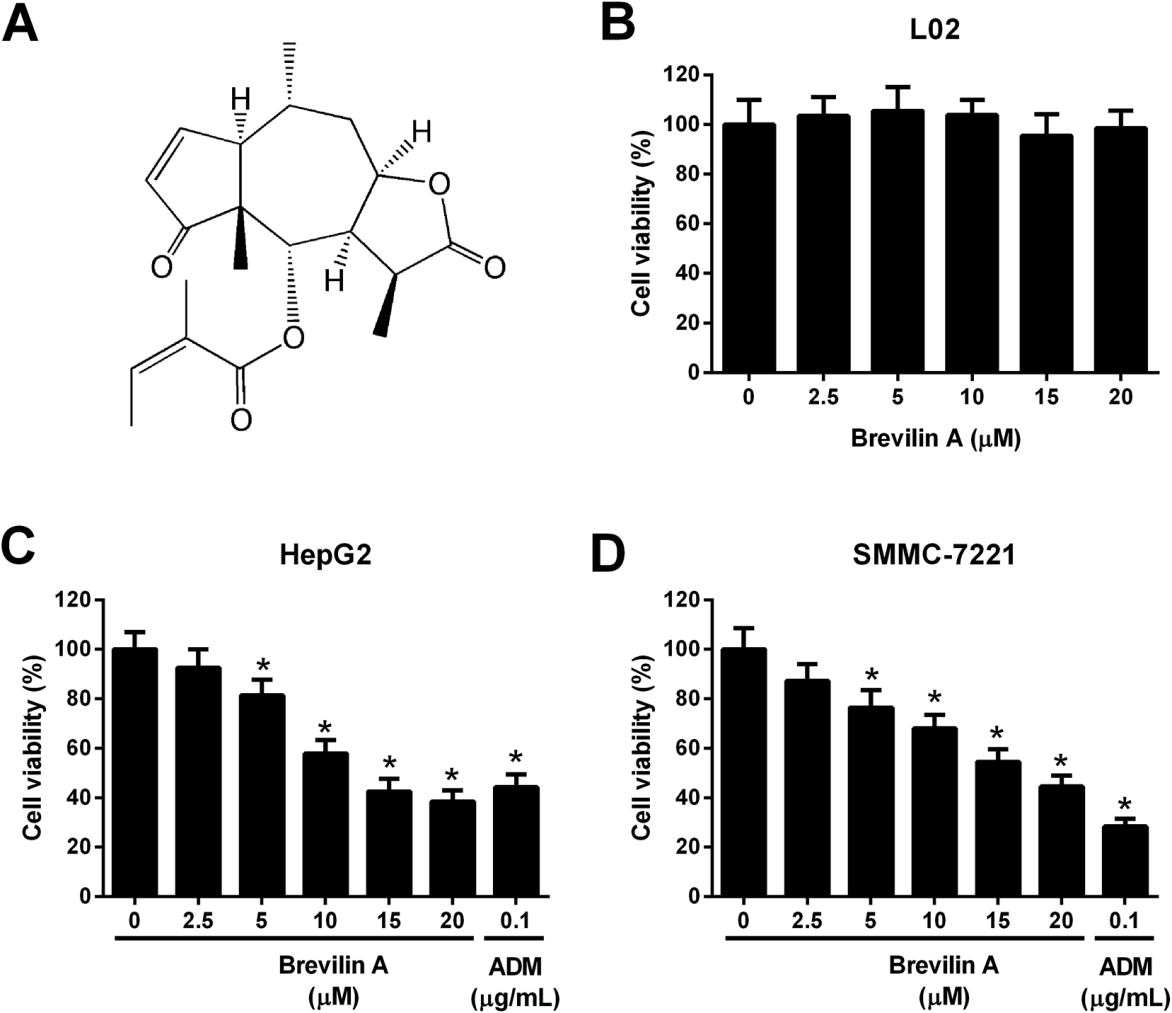
Brevilin A inhibits the viability of hepatoma cells. (A) Structure of brevilin A. (B) The normal liver cell line L02 was treated with brevilin A (0, 2.5, 5, 10, 15, or 20 μM) for 48 h. MTT assay was performed to detect the viability of L02 cells. (C and D) To evaluate the effect of brevilin A on cell viability, cells were treated with different concentrations of brevilin A (0, 2.5, 5, 10, 15, or 20 μM) for 48 h. MTT assay was performed to detect the viability of HepG2 and SMMC-7221 cells. Adriamycin (0.1 μg mL^−1^) was used as the positive control. *p < 0.05, significant relative to control cells.

*Centipeda minima* is a type of traditional Chinese herb that is commonly used for relieving stuffy nose, asthma, and cough.^9^ Pharmacological studies have demonstrated that the extracts from *Centipeda minima* possess various bioactivities including anti-bacterial, anti-allergic, anti-oxidant, and anti-inflammatory activities.^9–11^ Brevilin A (Fig. 1A) is a natural sesquiterpene lactone, which is firstly extracted from *Centipeda minima*.^12^ It has been reported that brevilin A displays anti-tumor effects against colon adenocarcinoma.^13^ However, the roles of brevilin A in HCC have not yet been reported. In the present study, we aimed to investigate the anti-tumor effect of brevilin A and the underlying mechanisms in HCC cell lines.

## 2. Materials and methods

### 2.1. Cell culture

The HCC cell lines HepG2 and SMMC-7221 and a normal liver cell line L02 were purchased from American Type Culture Collection (ATCC, Manassas, VA, USA). The cells were cultured in Dulbecco's modified Eagle medium (DMEM) supplemented with 10% fetal bovine serum (FBS), penicillin (100 U mL^−1^) and streptomycin (100 mg mL^−1^) and were maintained in atmosphere with 5% CO_2_ at 37 °C.

### 2.2. MTT assay

L02, HepG2, and SMMC-7221 cells were seeded in 96-well plates at a density of 0.5 × 10^4^ cells per well and incubated for 24 h. Brevilin A (purity > 98%; Yuanye Biotechnology, Shanghai, China) was dissolved in dimethyl sulfoxide (DMSO) to prepare a 5 mM stock solution. The culture media, containing brevilin A, were prepared by diluting the stock solution and the final DMSO concentration did not exceed 0.1%, which was nontoxic to L02, HepG2, and SMMC-7221 cells, as detected in our preliminary experiment. In order to evaluate the effect of brevilin A on the cell viability, the cells were treated with appointed doses of brevilin A for 48 h. Adriamycin (0.1 μg mL^−1^) was used as a positive control. Then, a 20 μL MTT (5 mg mL^−1^) was added to the cells and incubated for 4 h. Subsequently, the crystals were dissolved in 150 μL DMSO. The absorbance at 570 nm was measured with a microplate reader (Bio-Rad, Hercules, CA, USA). The 50% inhibitory concentration (IC_50_) values were calculated by the cell viability using GraphPad Prism 6.01 software (GraphPad Software Inc., San Diego, CA, USA).

### 2.3. Flow cytometry

For apoptosis assay, the HepG2 and SMMC-7221 cells were plated in 24-well plates. After incubation with 5 μM, 10 μM, or 15 μM brevilin A for 48 h, the cells were subjected to an Annexin V-PI dual staining using an Annexin V-FITC/PI Apoptosis Detection Kit (Beyotime Biotechnology, Shanghai, China). The apoptotic rate was detected using a BD FACSCalibur Flow Cytometer System (BD Biosciences, Franklin Lakes, NJ, USA).

### 2.4. Transwell assay

The invasive abilities of HepG2 and SMMC-7221 cells were measured using transwell assay with commercial chambers containing matrigel pre-coated inserts (Corning Inc., Corning, NY, USA). Cells in serum-free medium were added to the upper chamber, whereas the lower chamber was filled with normal medium cells. After incubating for 48 h, the invaded cells adhering to the lower side of the inserts were fixed with 4% paraformaldehyde, and then stained with crystal violet for 20 min. The numbers of the invaded cells were counted under an inverted microscope (Olympus, Tokyo, Japan).

### 2.5. Western blot

HepG2 and SMMC-7221 cells were lysed with RIPA lysis buffer (Beyotime) for 30 min at 4 °C. The contents of proteins in the whole cell lysates were determined by the BCA method (Thermo Fisher Scientific, Waltham, MA, USA). Equal quantities of proteins were then separated by 12% SDS-PAGE and subsequently transferred to PVDF membranes (Millipore, Billerica, MA, USA). Proteins were detected with indicated antibodies. The membranes were then blocked with bovine serum albumin (BSA) for 1 h, followed by incubation with primary antibodies at 4 °C overnight. The antibodies used in the study were anti-matrix metalloproteinase (MMP)-2 (Santa Cruz Biotechnology, Santa Cruz, CA, USA), anti-MMP-9 (Santa Cruz Biotechnology), anti-phospho-signal transducer and activator of transcription (p-Stat3) (Santa Cruz Biotechnology), anti-Stat3 (Santa Cruz Biotechnology), anti-Snail (Cell Signaling Technology), anti-β-catenin (Cell Signaling Technology), anti-c-Myc (Cell Signaling Technology), and anti-β-actin (Santa Cruz Biotechnology). The membranes were then incubated with HRP-conjugated secondary antibody (Cell Signaling Technology) diluted to 1 : 5000 in TBST at 37 °C for 2 h. The ECL detection reagent (Bio-Rad) was used for the visualization of the signals, according to the manufacturer's instructions.

### 2.6. Statistical analysis

All analyses were performed using SPSS statistical software (version 19.0, SPSS Inc., Chicago, IL, USA) with one-way analysis of variance (ANOVA) method. Error bars were used to represent standard deviation (SD). A *p*-value of <0.05 was considered statistically significant.

## 3. Results

### 3.1. Effect of brevilin A on the viability of hepatoma cells

To evaluate the effect of brevilin A on cell viability, L02, HepG2, and SMMC-7221 cells were treated with brevilin A (0 μM, 2.5 μM, 5 μM, 10 μM, 15 μM, or 20 μM) for 48 h. MTT assay showed that brevilin A at appointed concentrations did not significantly affect the viability of the normal liver cell line L02 (Fig. 1B). However, brevilin A inhibited the viability of HepG2 and SMMC-7221 cells in a dose-dependent manner with an IC_50_ value of 13.1 μM and 17.7 μM, respectively. Adriamycin treatment (positive control) also suppressed the viability of HepG2 and SMMC-7221 cells (Fig. 1C and D). An increase in brevilin A concentrations to 15 μM did not show an outstanding increase in the inhibitory effect on viability; therefore, concentrations of 5 μM, 10 μM, and 15 μM were selected for the following experiments.

### 3.2. Effect of brevilin A on apoptosis of hepatoma cells

**Fig. 2:**
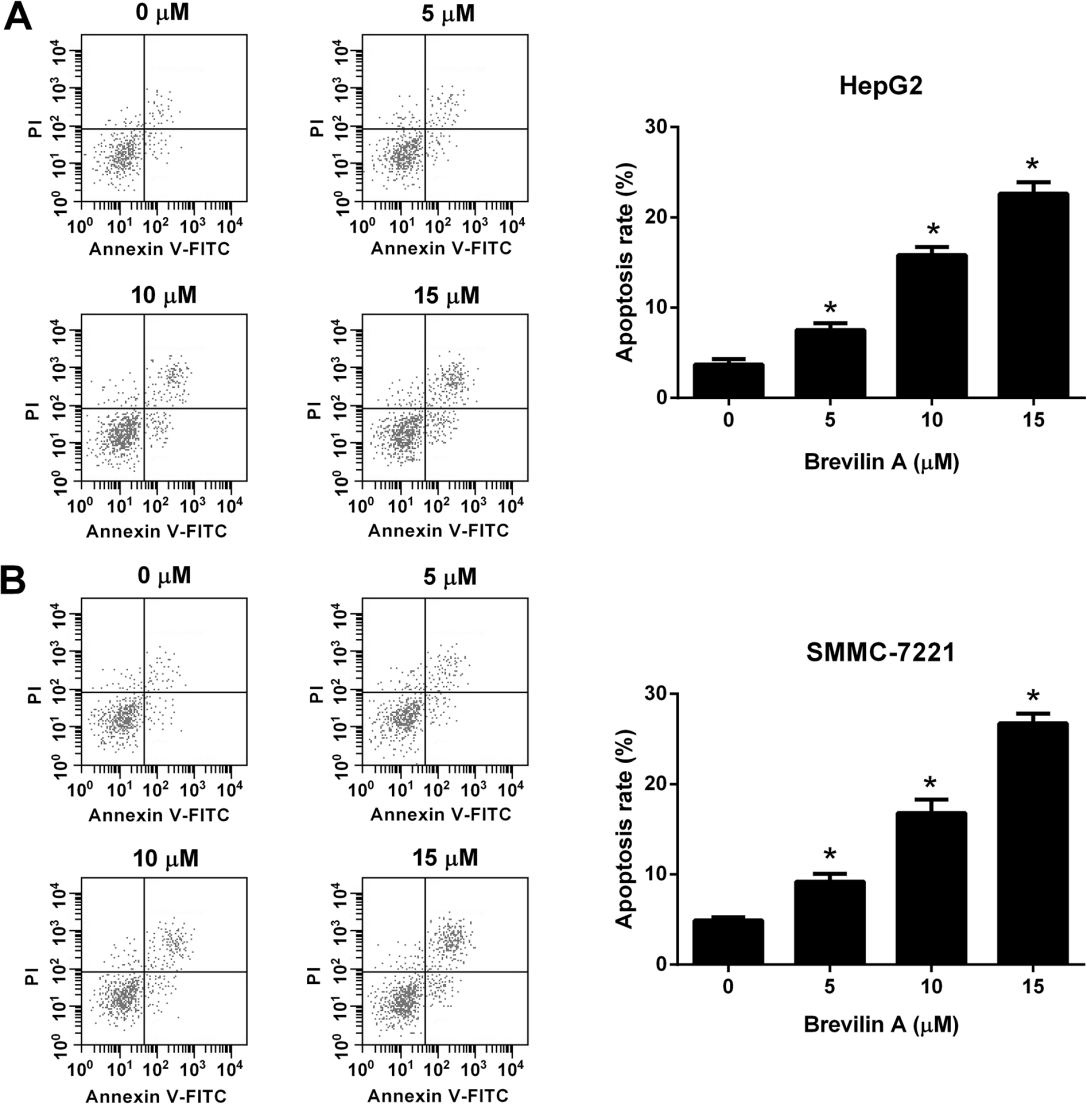
Brevilin A induces cell apoptosis of hepatoma cells. Cells were treated with different concentrations of brevilin A (0, 5, 10, or 15 μM) for 48 h. The flow cytometry was performed to detect cell apoptosis of HepG2 (A) and SMMC-7221 cells (B). *p < 0.05, significant relative to control cells.

We next investigated the effect of brevilin A on apoptosis of HepG2 and SMMC-7221 cells. After incubating with brevilin A (5 μM, 10 μM, or 15 μM) for 48 h, the flow cytometry was performed to detect cell apoptosis. As shown in Fig. 2A, the apoptotic rate of HepG2 cells treated with brevilin A (5 μM, 10 μM, or 15 μM) was increased compared to the control cells. Brevilin A (5 μM, 10 μM, or 15 μM) also elevated the apoptotic rate of SMMC-7221 cells (Fig. 2B).

### 3.3. Effect of brevilin A on invasion of hepatoma cells

**Fig. 3:**
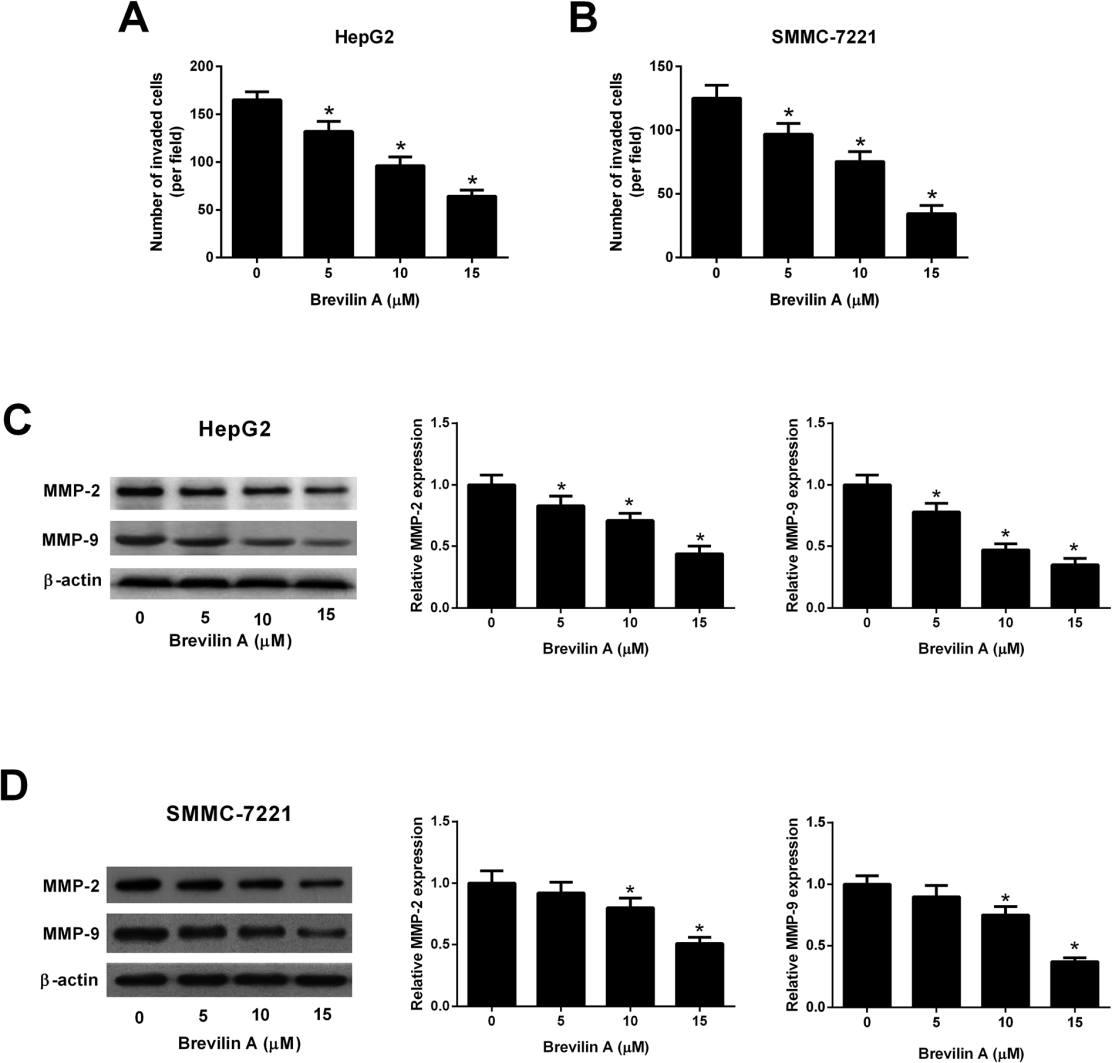
Brevilin A suppresses cell invasion of hepatoma cells. After incubation with different concentrations of brevilin A (0, 5, 10, or 15 μM) for 48 h, cell invasion of HepG2 (A) and SMMC-7221 cells (B) was detected using transwell assay. *p < 0.05, significant relative to control cells. (C and D) Western blot analysis was performed to determine the expressions of MMP-2 and MMP-9 in HepG2 and SMMC-7221 cells. *p < 0.05, significant relative to control cells.

The transwell assay was performed to measure the effect of brevilin A on cell invasion. The number of invaded HepG2 cells were significantly decreased after the treatment with brevilin A (5 μM, 10 μM, or 15 μM), indicating that brevilin A caused a lower invasive ability in HepG2 cells (Fig. 3A). Moreover, the invasive ability of SMMC-7221 cells was also inhibited by brevilin A (5 μM, 10 μM, or 15 μM) (Fig. 3B). The western blot analysis was performed to evaluate the effect of brevilin A on MMP expressions. As shown in Fig. 3C, the expression levels of MMP-2 and MMP-9 were decreased in HepG2 cells treated with brevilin A (5 μM, 10 μM, or 15 μM), as compared to the control cells. In addition, the MMP-2 and MMP-9 expressions were also suppressed by brevilin A (5 μM, 10 μM, or 15 μM) in SMMC-7221 cells (Fig. 3D).

### 3.4. Effect of brevilin A on the STAT3/Snail pathway

**Fig. 4:**
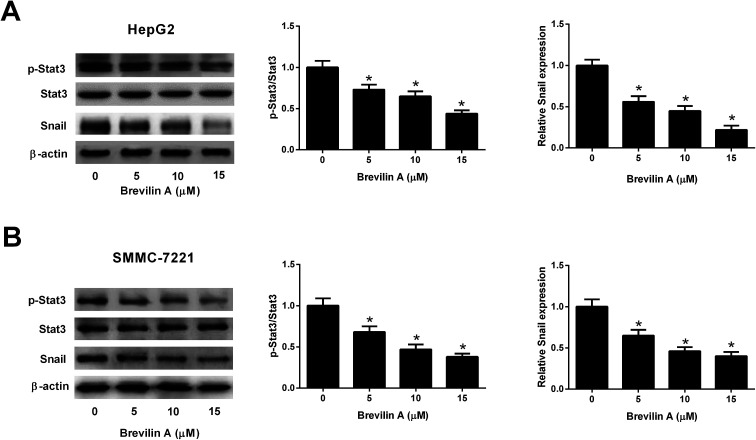
Brevilin A blocks the STAT3/Snail pathway in hepatoma cells. To investigate the effect of brevilin A on STAT3/Snail pathway, HepG2 and SMMC-7221 cells were treated with different concentrations of brevilin A (0, 5, 10, or 15 μM) for 48 h. The expressions of p-STAT3, STAT3 and Snail in HepG2 (A) and SMMC-7221 cells (B) were detected using western blot analysis. *p < 0.05, significant relative to control cells.

Further experiments were performed to investigate the mechanism of the bioactivities of brevilin A in hepatoma cells. The western blot analysis showed that the expressions of p-STAT3 and Snail were reduced after treating them with brevilin A (5 μM, 10 μM, or 15 μM) in both HepG2 and SMMC-7221 cells (Fig. 4A and B). However, the expression level of STAT3 was not altered. The results indicated that brevilin A suppressed the STAT3/Snail pathway in hepatoma cells.

### 3.5. Effect of brevilin A on the Wnt/β-catenin pathway

**Fig. 5:**
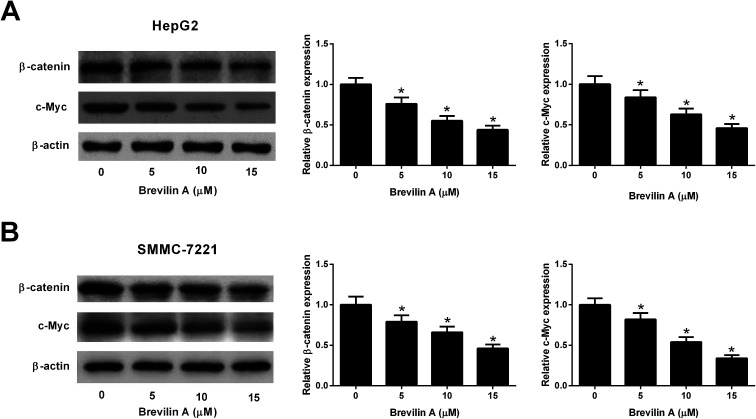
Brevilin A suppresses the Wnt/β-catenin pathway in hepatoma cells. The effect of brevilin A on the Wnt/β-catenin pathway was explored by detecting the expressions of β-catenin and c-Myc in HepG2 (A) and SMMC-7221 cells (B) using western blot analysis after incubation with different concentrations of brevilin A (0, 5, 10, or 15 μM) for 48 h. *p < 0.05, significant relative to control cells.

We also explored the effect of brevilin A on the Wnt/β-catenin pathway by detecting the expressions of β-catenin and c-Myc using the western blot analysis. The results shown in Fig. 5A and B revealed that brevilin A resulted in a decrease in the expressions of β-catenin and c-Myc in both HepG2 and SMMC-7221 cells in a dose-dependent manner. These findings suggested that brevilin A exerted inhibitory effect on the Wnt/β-catenin pathway in the hepatoma cells.

## 4. Discussion

*Centipeda minima* is a Chinese herbal medicine that is usually used in the treatment of various diseases. In recent years, *Centipeda minima* has been found to possess broad activities, which are attributed to the main bioactive constituents, sesquiterpene lactones.^14^ Huang *et al.*^14^ showed that sesquiterpene lactones from *Centipeda minima* display anti-angiogenic activity. It is well known that angiogenesis is essential for tumor growth and metastasis; therefore, sesquiterpene lactones may be considered as a promising candidate for preventing the tumor development and progression though inhibiting angiogenesis.^14^ 6-*O*-Angeloylenolin (6-OA), a sesquiterpene lactone isolated from *Centipeda minima*, exerts anti-tumor effect against lung cancer.^15^ You *et al.*^13^ reported that brevilin A exhibits anti-tumor activity against colon adenocarcinoma *via* inducing apoptosis and autophagy of CT26 cells *via* mitochondrial and PI3K/AKT/mTOR pathways. Chen *et al.*^16^ demonstrated that brevilin A is a potential inhibitor of Janus kinase (JAK) activity and STAT signaling in cancer cells, suggesting that brevilin A may be a therapeutic agent for the cancer patients with hyperactivated JAKs and STATs. However, researches on the bioactivity of brevilin A in HCC have not yet been reported.

In the present study, we investigated the anti-tumor effect of brevilin A against HCC *in vitro*. The results showed that brevilin A reduced cell viability and invasion, and induced cell apoptosis of HCC cells. Extracellular matrix (ECM) is a group extracellular proteins that provide structural and biochemical support to the surrounding cells.^17^ The ECM plays crucial roles in cancer progression, invasion and metastasis, and deregulation of the ECM dynamics is a hallmark of cancer.^17^ The MMP is a family of proteolytic enzymes that are responsible for the degradation of the multiple components of the ECM.^18^ A large body of experimental and clinical evidence has implicated that targeting MMPs might be reconsidered for the cancer therapy.^18^ Our results also revealed that brevilin A inhibited the expressions of MMP-2 and MMP-9 in HCC cells. Taken together, brevilin A exhibited anti-tumor activity against HCC; however, further *in vivo* investigations are needed in the future studies.

Snail is a well-characterized repressor of E-cadherin, which is required for triggering epithelial–mesenchymal transition (EMT) and tumor metastasis.^19^ In many types of cancers, Snail expression is regulated by Stat3 signaling pathway that plays a key role in many cellular processes, such as cell growth and apoptosis.^19^ Previous studies proved that the Stat3/Snail signaling pathway is activated in HCC. Inhibition of the Stat3/Snail signaling pathway might be a promising therapeutic strategy to reduce the aggressiveness of HCC cells.^20^ Brevilin A was found to inhibit the STAT3 signaling in human lung cancer A549 cells.^16^ In the current study, we found that brevilin A suppressed the phosphorylation of STAT3 and expression of Snail in HCC cells, indicating that brevilin A inactivated the Stat3/Snail signaling pathway.

The Wnt/β-catenin signaling is an important pathway involved in several physiological and pathological processes, thereby participating in embryonic development and carcinogenesis.^21^ It has been denoted that Wnt/β-catenin signaling plays a critical role in liver development, liver regeneration, and liver zonation.^22,23^ Furthermore, aberrant activation of this signaling pathway has been found in HCC, and has been correlated with tumor progression and poor prognosis.^22^ Thus, Wnt/β-catenin pathway is considered as a potential application for the treatment of HCC.^22^ We found that brevilin A reduced the expressions of β-catenin and c-Myc, which are two important members in the Wnt/β-catenin pathway. The results suggested that brevilin A blocked the activation of the Wnt/β-catenin pathway in HCC cells.

## 5. Conclusion

In conclusion, our data provided a new insight into the anti-tumor effect of brevilin A against HCC *in vitro*. The Stat3/Snail and Wnt/β-catenin signaling pathways are involved in the effects of brevilin A on HCC cells. We speculated that brevilin A would be a potential therapeutic agent to control the development and progression of HCC.

## Conflicts of interest

The authors declare that they have no conflict of interest.

## Supplementary Material
